# Mediation of circ_RPPH1 on miR-146b-3p/E2F2 pathway to hinder the growth and metastasis of breast carcinoma cells

**DOI:** 10.18632/aging.203439

**Published:** 2021-08-25

**Authors:** Hai Feng, Shou-Zhan Sun, Fang Cheng, Nian-Qu Zhang

**Affiliations:** 1 Department of Anesthesiology, The First Affiliated Hospital of Shandong First Medical University, Shandong Provincial Qianfoshan Hospital, Jinan, Shandong 250014, China; 2 Department of Anesthesiology, West Hospital, Qingdao Municipal Hospital, Qingdao, Shandong, China; 3 Clinical Laboratory, The First Affiliated Hospital of Shandong First Medical University, Shandong Provincial Qianfoshan Hospital, Jinan, Shandong 250014, China; 4 Department of Breast Surgery, The First Affiliated Hospital of Shandong First Medical University, Shandong Provincial Qianfoshan Hospital, Jinan, Shandong 250014, China

**Keywords:** E2F2, breast carcinoma, circ_RPPH1, miR-146b-3p

## Abstract

Background: Nova Circular RNA (circRNA) of non-coding RNA has gradually become an important regulatory factor, and it has made people attach great concern over the occurrence and development of many diseases, particularly carcinomas. circ_RPPH1 is a newly discovered circRNA. Gene Expression Omnibus (GEO) analysis showed that there are high contents of circ_RPPH1 in breast cancer (BC), but the mechanism of circRNA in BC remains unclear.

Methods: Real-time quantitative PCR (qRT-PCR) was applied to test the role of circ_RPPH1 in BC patients, and functional experiments were applied to test the role of circ_RPPH1 on BC tumor. Fluorescence *in situ* hybridization, double luciferase reporter gene analysis, RNA pull-down and RNA immunoprecipitation experiments were performed to explore the correlation of circ_RPPH1 with miR-146b-3p/E2F2 in BC.

Results: circ_RPPH1 was evidently enhanced in BC, and its content was related to the clinical stage and pathological grade. circ_RPPH1 can accelerate the proliferation, migration and invasion, and promote tumorigenesis and metastasis. Mechanism exploration indicated that circ_RPPH1 acted as ceRNA (competing endogenous RNA) of miR-146b-3p, so as to reduce the inhibitory role of miR-146b-3p on its target E2F2.

Conclusion: Circ_RPPH1/miR-146b-3p/E2F2 axis can promote the progression of BC, and it might be a latent therapeutic target for clinical BC.

## INTRODUCTION

Breast carcinoma (BC) has been recognized as the most common malignant tumor and the main cause of carcinoma death in women worldwide [1, 2]. Although progresses have been made in the treatment of BC, the metastasis, recurrence and drug resistance caused by long-term treatment of chemotherapy drugs have not been evidently improved, which is also the issue that puzzles the clinical treatment [3–5]. Therefore, exploring the molecular mechanism of BC to develop more effective treatment strategies is urgent.

The development mechanism of BC is complex, which includes the functions of various genetic and epigenetic factors [6, 7]. Studies have shown that non-coding RNA has a correlation with the human life process and can regulate many diseases, especially tumors [8]. MicroRNA (miR) and long non-coding RNA (LncRNA) have become hot research fields [9, 10]. Circular RNA (circRNA) is a newly discovered covalently closed continuous loop, in which 3′ and 5′RNA ends are connected, and there is no polyadenylic acid counterpart, so it has higher stability than linear type [11–13]. However, its exact role in human diseases is still unclear and needs further exploration [14]. CircRNA, the same as lncRNA, acts in the molecular level as a "sponge" of miR and absorbs functional miR, thus reducing the abundance of miRNA in cytoplasm and regulating the expression of miR target gene [15–17]. Circ_RPPH1, also known as hsa_circ_0000517, is located on chr14:20811404-20811492. Early explorations have found that circ_RPPH1 is enhanced in hepatocellular carcinoma, which can play a carcinogenic role by regulating miR-1296-5p [18]. However, in this study, we analyzed GSE101123 chip and found that circ_RPPH1 is highly expressed in BC, which might be a latent target for BC.

Therefore, we tested the role of circ_RPPH1 in BC by analyzing human circRNA chip (GSE101123).

## RESULTS

### Circ_RPPH1 is highly expressed in BC

**Figure 1:**
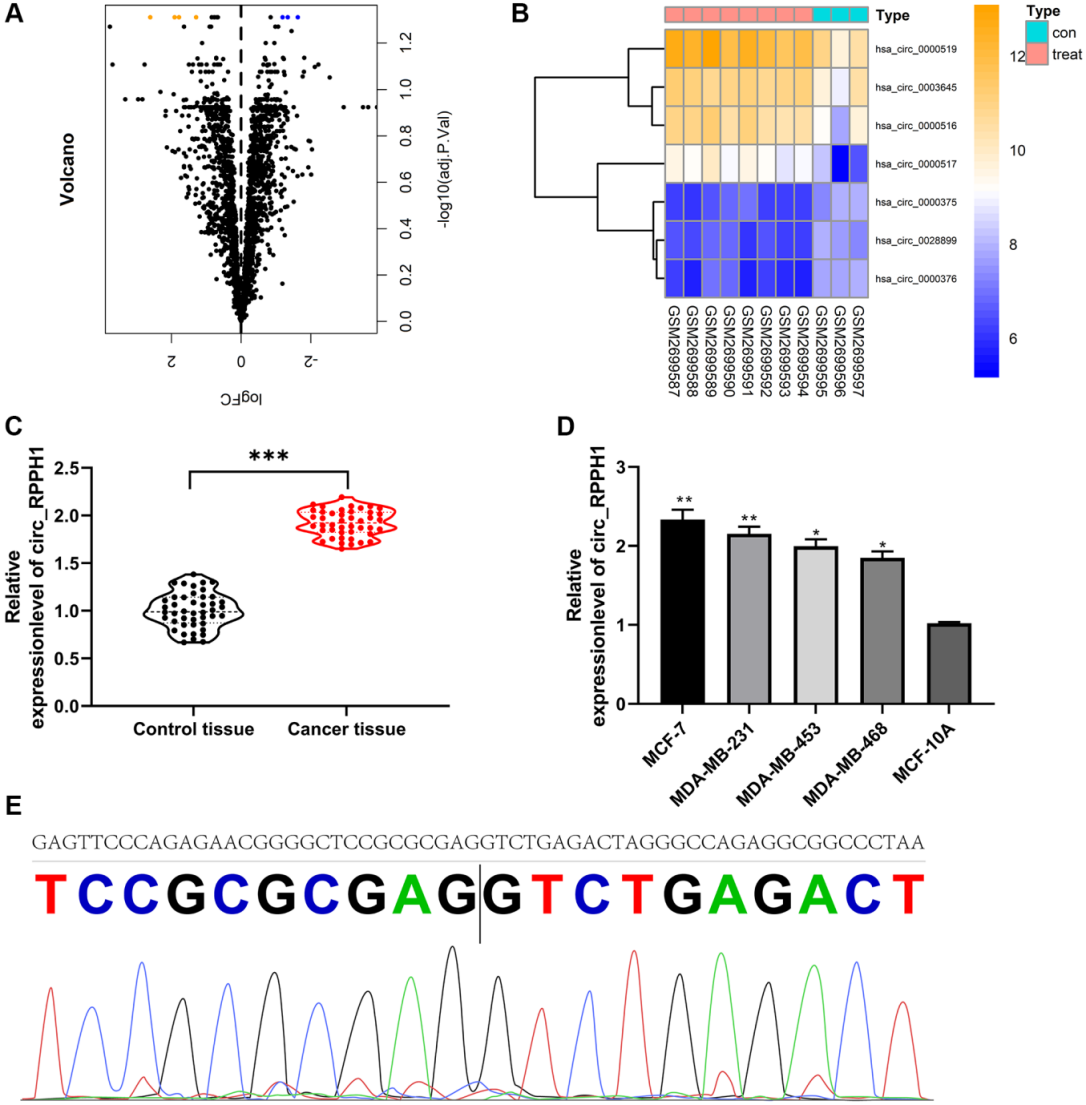
**Expression of circ_RPPH1 in BC.** (**A**) limma package analysis of differential circRNA volcanic map in GSE101123 chip. (**B**) Visualizing of differential circRNA heat map by pheatmap package. (**C**) qRT-PCR was used to detect the relative expression of circ_RPPH1 in BC patients. (**D**) The relative expression of circ_RPPH1 in BC cell lines was detected by qRT-PCR. (**E**) The sequence of circ_RPPH1 in circBase (upper part) is consistent with that in Sanger sequencing (lower part). ^*^indicates *P* < 0.05; ^**^indicates *P* < 0.01; ^***^indicates *P* < 0.001.

At first, we analyzed the circRNA with differences in GSE101123 chip. Through limma package, we selected 7 circRNAs with differences in expression, including 4 CIRC RNAs with high contents and 3 CIRC RNAs with low contents (Figure 1A, 1B, Table 1). According to the logFC of different circRNA, the largest hsa_circ_0000517 (circ_RPPH1) was selected for research. circ_RPPH1 in BC was evidently elevated than that in adjacent tissues (Figure 1C). Furthermore, according to the median expression of circ_RPPH1, the patients were grouped into high and low expression groups. Patients with high circ_RPPH1 had high TNM score (III + IV), and the probability of lymphatic metastasis increased evidently (Table 2). In addition, it was found that circ_RPPH1 was elevated in BC cell lines by qRT-PCR (Figure 1D). Finally, we confirmed that circ_RPPH1 sequence was consistent with that in circBase by Sanger sequencing (Figure 1E). These results indicated that circ_RPPH1 was expected to be a potential indicator of BC.

**Table 1:** Differential circRNA expression.

**Gene**	**logFC**	**AveExpr**	**t**	***P*.Value**	**β**
hsa_circ_0000376	–1.628	6.616	–5.699	1.160E-04	1.497
hsa_circ_0000375	–1.339	6.771	–5.571	1.412E-04	1.320
hsa_circ_0028899	–1.188	6.734	–5.891	8.670E-05	1.759
hsa_circ_0003645	1.291	10.638	5.256	2.318E-04	0.871
hsa_circ_0000516	1.785	10.257	5.176	2.632E-04	0.755
hsa_circ_0000519	1.913	11.747	5.620	1.310E-04	1.388
hsa_circ_0000517	2.611	8.540	5.390	1.873E-04	1.064

**Table 2:** circ_RPPH1 and clinical data analysis of BC patients.

**Factor**		**circ_RPPH1**		***P* value**
		**High expression (*n* = 22)**	**Low expression (*n* = 22)**	
Age				0.540
	≥50 (*n* = 18)	10	8
	<50 (*n* = 26)	12	14
Tumor size				0.353
	≥2cm (*n* = 17)	7	10
	<2cm (*n* = 27)	15	12
Menopausal status				0.761
	Menopause (*n* = 19)	9	10
	Non-menopause (*n* = 25)	13	12
TNM staging				0.015
	I + II (*n* = 24)	8	16
	III + IV (*n* = 20)	14	6
Lymphatic metastasis				0.021
	Transfer (*n* = 13)	10	3
	Not transferred (*n* = 31)	12	19
ER				0.555
	Negative (*n* = 23)	11	12
	Positive (*n* = 21)	13	10
PR				0.361
	Negative (*n* = 19)	8	11
	Positive (*n* = 25)	14	11
HER2				0.540
	Negative (*n* = 18)	8	10
	Positive (*n* = 26)	14	12

### Down-regulating the expression of circ_RPPH1 can hinder the development and metastasis of BC cells

**Figure 2:**
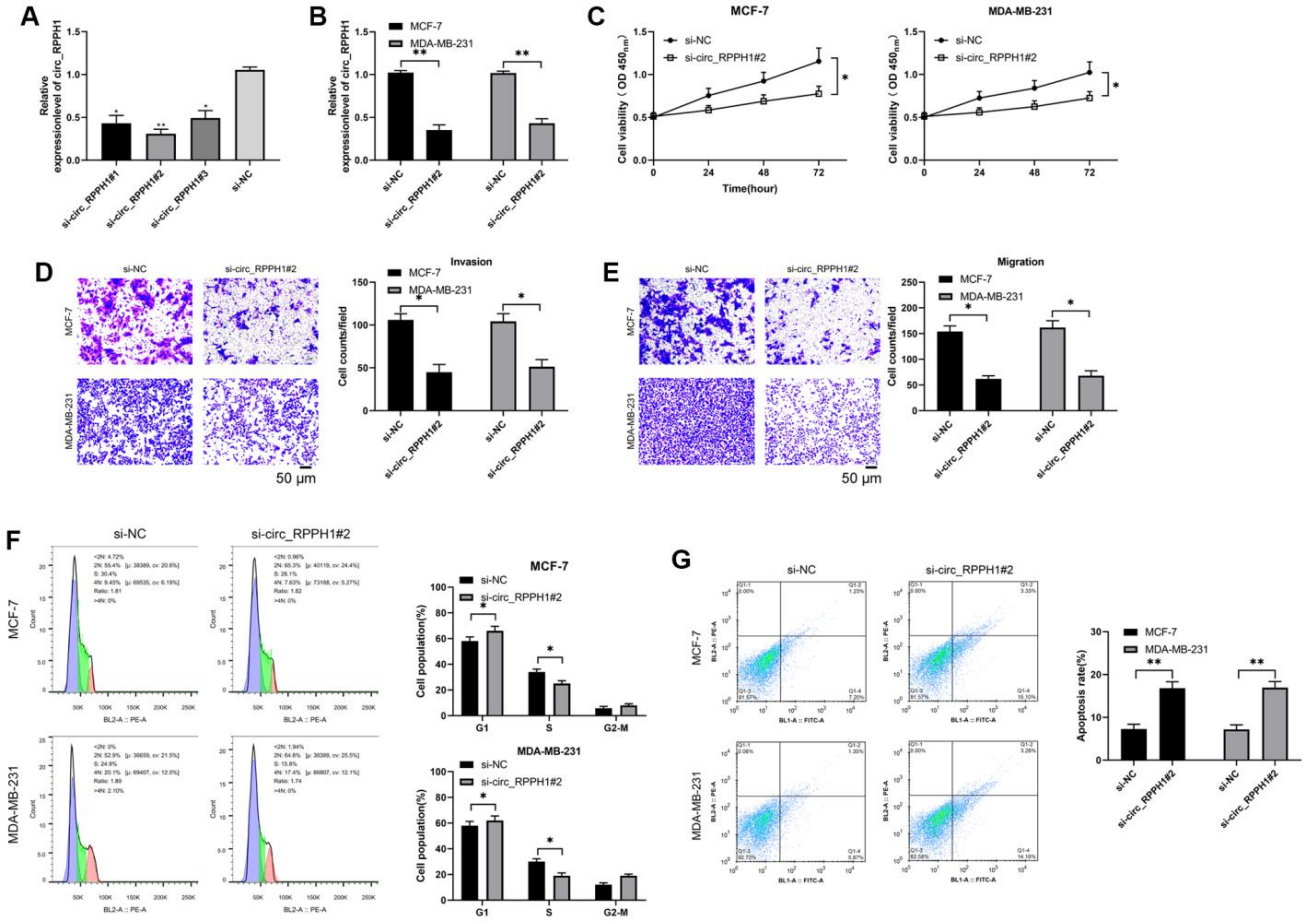
**Carcinogenic role of circ_RPPH1 in BC.** (**A**) qRT-PCR was used to detect the relative expression of circ_RPPH1 in si-circ_RPPH1. (**B**) qRT-PCR was used to detect the relative expression of circ_RPPH1 in BC cells transfected with si-circ_RPPH1#2. (**C**) CCK-8 test was used to detect the proliferation of BC cells transfected with si-circ_RPPH1#2. (**D**, **E**) Transwell test showed the invasion and migration of BC cells transfected with si-circ_RPPH1#2. Scale bars, 50 μm. (**F**, **G**) The changes of cell cycle and apoptosis rate of BC cells transfected with si-circ_RPPH1#2 were detected by flow cytometry. ^*^indicates *P* < 0.05; ^**^indicates *P* < 0.01.

It was concluded that circ_RPPH1 was enhanced in BC. RNase R assays demonstrated that the linear mRNA RPPH1 was digested, while circ_RPPH1 not (Supplementary Figure 1A). Actinomycin D assay shown that circ_RPPH1 was more stable compared with linear RPPH1 (Supplementary Figure 1B). In order to further confirm the anti-carcinoma role of circ_RPPH1 in BC, we first established three siRNA, and selected si-circ_RPPH1#2 with the most significant difference (Figure 2A), which was transfected into MCF-7 and MDA-MB-23 respectively (Figure 2B), and the transfection efficiency was determined by qRT-PCR. Then we further detected the cell growth and metastasis by CCK-8, Transwell and flow cytometry. Compared with si-NC, the proliferation of BC cells transfected with si-circ_RPPH1#2 was evidently hindered (Figure 2C). The invasion and migration of BC cells transfected with si-circ_RPPH1#2 were also evidently hindered (Figure 2D, 2E). This indicated that transfection of si-circ_RPPH1#2 can slow down cell metastasis by inhibiting cell proliferation. In addition, we also found that transfection of si-circ_RPPH1#2 can obviously block S-phase BC cells, thus inducing the apoptosis of BC cells (Figure 2F, 2G). These experiments showed that circ_RPPH1 downregulation could hinder the growth and metastasis of BC and was expected to be a potential therapeutic target.

Moreover, we validated the efficiency of circ_RPPH1 overexpression in BC cells (Supplementary Figure 2A). The proliferation of BC cells was promoted by circ_RPPH1 overexpression (Supplementary Figure 2B and 2C). The invasion and migration of BC cells were induced by circ_RPPH1 overexpression (Supplementary Figure 2D and 2E). The overexpression of circ_RPPH1 inhibited G1-phase BC cells and enhanced S-phase BC cells (Supplementary Figure 2F and 2G). In addition, the apoptosis of BC cells was repressed by circ_RPPH1 overexpression (Supplementary Figure 2H and 2I). Together these data confirmed that circ_RPPH1 promote the growth and metastasis of BC.

### circ_RPPH1 can act as miR-146b-3p sponge

**Figure 3:**
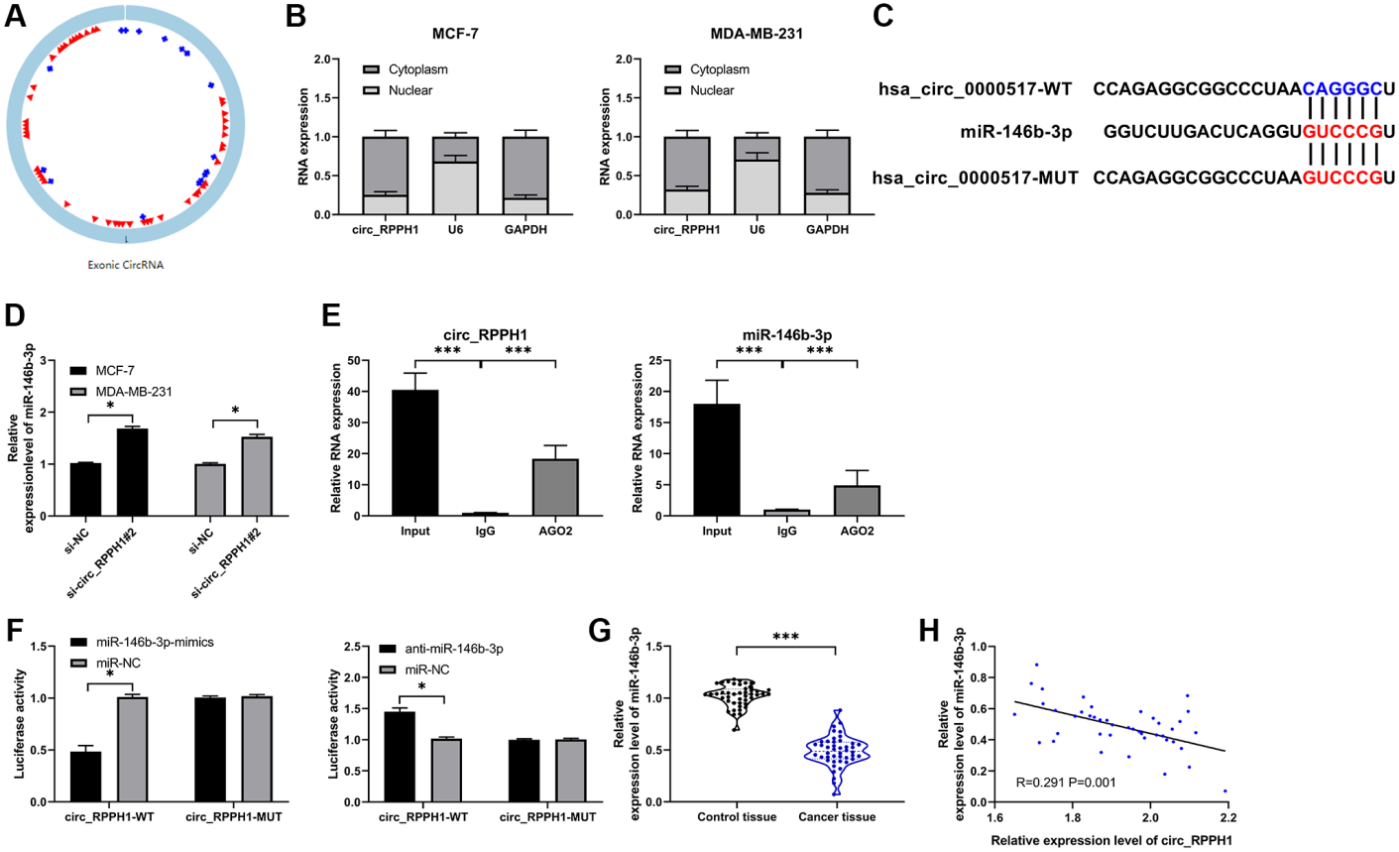
**circ_RPPH1 acts as miR-146b-3p sponge for regulation.** (**A**) circ_RPPH1 structure. (**B**) Subcellular localization analysis of circ_RPPH1 distribution in MCF-7 and MDA-MB-231 cells. (**C**) Circinteractome predicted the target site between circ_RPPH1 and miR-146b-3p. (**D**) The relative expression of miR-146b-3p in cells transfected with si-circ_RPPH1#2 was detected by qRT-PCR. (**E**) RIP test detected the targeted binding of circ_RPPH1 and miR-146b-3p. (**F**) Double luciferase report experimental analysis of targeted binding between circ_RPPH1 and miR-146b-3p. (**G**) qRT-PCR was used to detect the expression of miR-146b-3p in BC patients. (**H**) Pearson test was applied to analyze the correlation between miR-146b-3p and circ_RPPH1 in tumor tissues of BC patients. ^*^indicates *P* < 0.05; ^**^indicates *P* < 0.01.

The sponge role of circRNA on miR depends on its location in cells (Figure 3A). In order to determine the distribution of circ_RPPH1, we analyzed MCF-7 and MDA-MB-231 respectively. Fluorescence *in situ* hybridization showed that circ_RPPH1 was mainly distributed in cytoplasm of the two (Figure 3B and Supplementary Figure 3A). In addition, subcellular localization analysis also found that the content of circ_RPPH1 in the cytoplasm of the two was evidently higher than that in the nucleus. Therefore, circ_RPPH1 has the potential to regulate miR. Therefore, we predicted the potential miR of circ_RPPH1 through the online prediction website of circinteractome (https://circinteractome.irp.nia.nih.gov/), [19] and found that there is a targeted binding between circ_RPPH1 and miR-146b-3p (Figure 3C and Supplementary Figure 3B). To confirm this binding, we tested miR-146b-3p in BC cells transfected with si-circ_RPPH1#2 by qRT-PCR, and found that miR-146b-3p in cells transfected with si-circ_RPPH1#2 enhanced evidently (Figure 3D), suggesting that circ_RPPH1 can regulate miR-146b-3p. Then we confirmed that circ_RPPH1 acts as miR-146b-3p sponge by RIP test and double luciferase report (Figure 3E, 3F). We also tested miR-146b-3p in BC patients by qRT-PCR, and found that miR-146b-3p in BC patients decreased evidently (Figure 3G). Correlation analysis showed that miR-146b-3p had a negative correlation with circ_RPPH1 (Figure 3H), and circ_RPPH1 could regulate miR-146b-3p.

### miR-146b-3p targeted E2F2

**Figure 4:**
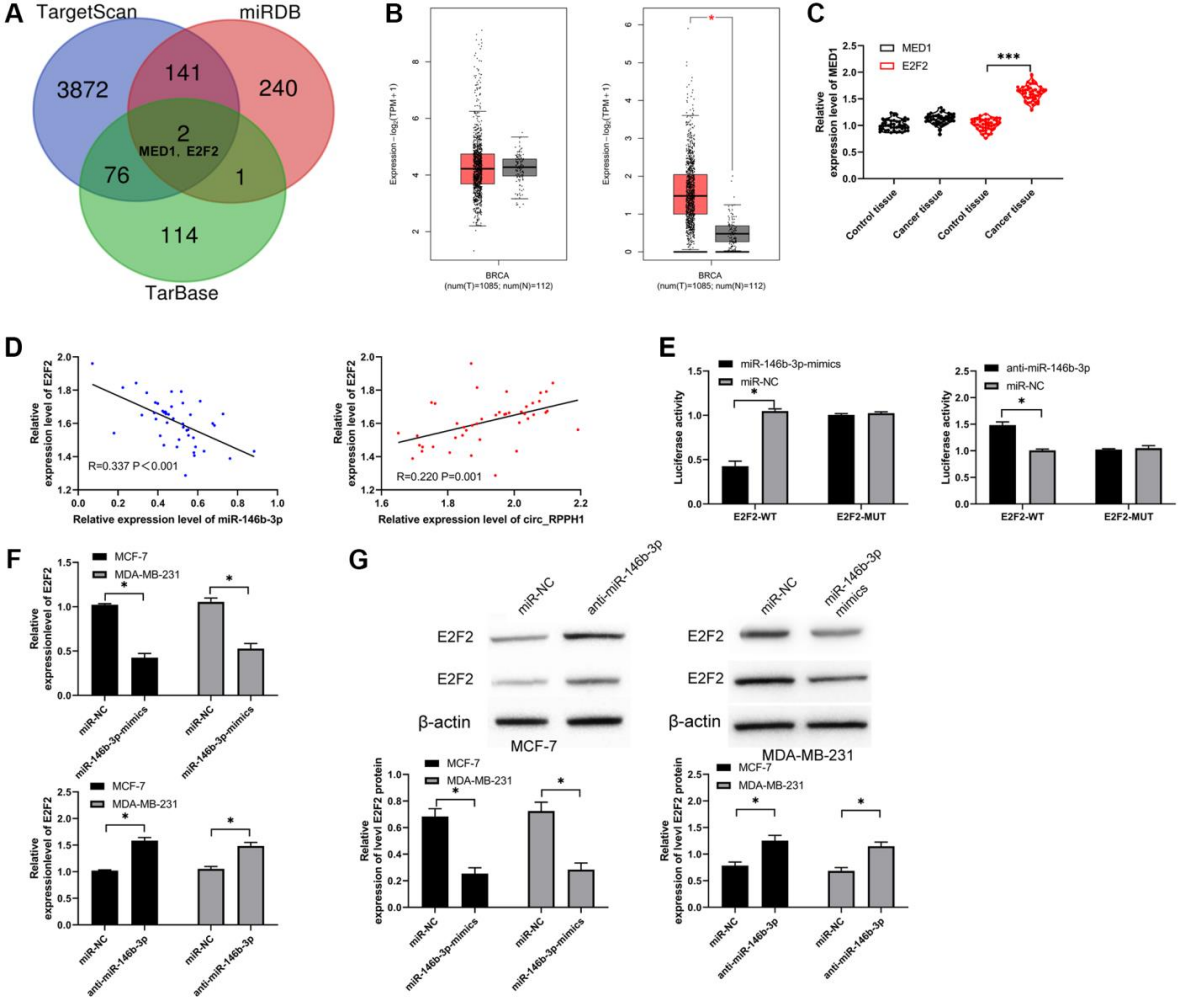
**miR-146b-3p targets E2F2 and regulates it.** (**A**) Three target genes prediction websites predicted the potential common target gene of miR-146b-3p. (**B**) GEPIA2 analyzed the relative expression of MED1 and E2F2 in BC tissues in TCGA database. (**C**) qRT-PCR was used to detect the relative expression of MED1 and E2F2 in BC patients. (**D**) Pearson test was used to analyze the correlation among circ_RPPH1, miR-146b-3p and E2F2 in tumor tissues of BC patients. (**E**) Double luciferase report analysis of miR-146b-3p binding to E2F2. (**F**, **G**) The relative expression of E2F2 in BC cell lines transfected with miR-146b-3p-mimics and anti-miR-146b-3p was detected by qRT-PCR and WB. ^*^indicates *P* < 0.05; ^***^indicates *P* < 0.001.

We predicted the targets through TaragetScan, [20] miRDB [21] and TarBase [22] to explore the downstream target gene of miR-146b-3p (Figure 4A). As a result, we found two common potential target genes (MED1 and E2F2). We analyzed MED1 and E2F2 through online GEPIA2 [23] software, and found that MED1 had no significant difference in BC, but E2F2 was highly expressed in BC (Figure 4B). Subsequently, we also found that there was no difference in MED1 in BC tissues compared with adjacent tissues by qRT-PCR, while E2F2 increased in BC tissues (Figure 4C). Furthermore, the correlation analysis suggested that E2F2 had a negative correlation with miR-146b-3p and a positive correlation with circ_RPPH1, suggesting that miR-146b-3p was targeted to E2F2 (Figure 4D). To verify this, we found that miR-146b-3p-mimics can hinder the fluorescence activity of E2F2-WT, while anti-miR-146b-3p can promote the fluorescence activity (Figure 4E). In addition, qRT-PCR and WB detection also found that E2F2 mRNA and protein were evidently hindered after transfection of miR-146b-3p-mimics and promoted after transfection of anti-miR-146b-3p (Figure 4F, 4G). Therefore, miR-146b-3p can regulate E2F2 in a targeted manner.

### circ_RPPH1 promoted E2F2 to regulate the growth and metastasis of BC cells by down-regulating miR-146b-3p

**Figure 5:**
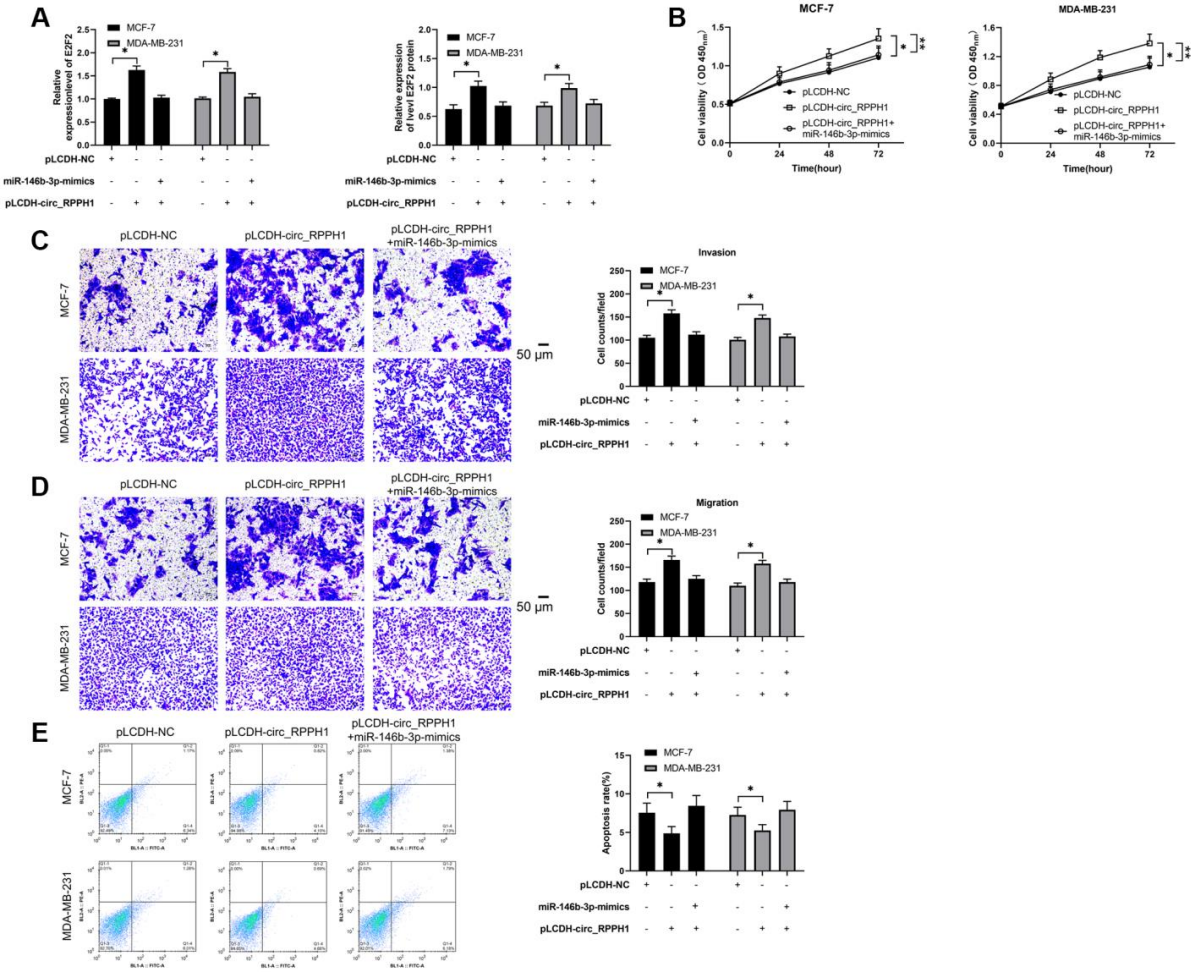
**circ_RPPH1/miR-146b-3p/E2F2 regulates the growth and metastasis of BC cells.** (**A**) qRT-PCR and WB test were used to detect the relative expression of E2F2 mRNA and protein in co-transfected cells. (**B**) CCK-8 experiment was used to detect the proliferation changes of cells after co-transfection. (**C**, **D**) Transwell test was used to detect the changes of cell invasion number and migration number after co-transfection. Scale bars, 50 μm. (**E**) The change of apoptosis rate after co-transfection was detected by flow cytometry. ^*^indicates *P* < 0.05.

To verify that circ_RPPH1 can regulate miR-146b-3p/E2F2 axis, we first performed co-transfection. qRT-PCR and WB detection showed that E2F2 mRNA and protein in cells transfected with pLCDH-circ_RPPH1 increased obviously, but there was no evident difference in vector after co-transfection of pLCDH-circ_RPPH1 and miR-146b-3p-mimics (Figure 5A). Therefore, circ_RPPH1 can regulate E2F2 by acting as miR-146b-3p sponge. Then, we also tested the biological function of the cells after co-transfection. Through CCK-8, Transwell and flow cytometry experiments, we found that the proliferation of cells (Figure 5B), the number of invasion (Figure 5C) and migration (Figure 5D), and the inhibition of apoptosis (Figure 5E) was reversed by co-transfection of miR-146b-3p-mimics. Therefore, circ_RPPH1 can promote apoptosis through miR-146b-3p/E2F2 axis and hinder the growth and metastasis of BC cells.

### Inhibition of circ_RPPH1 can improve tumor growth in nude mice with BC

**Figure 6:**
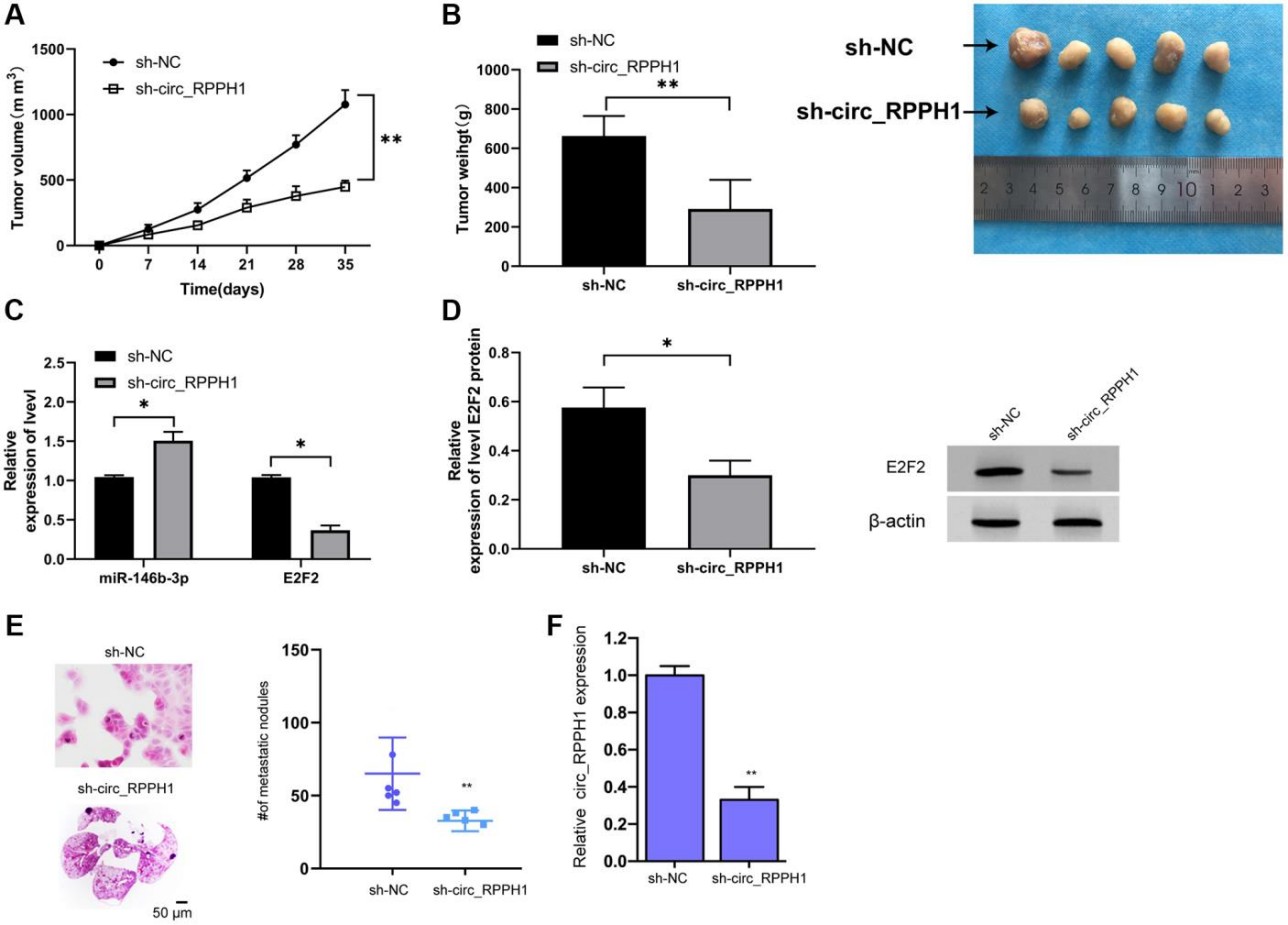
**circ_RPPH1 can improve tumor growth in nude mice with BC.** (**A**) Tumor growth in nude mice within 5 weeks. (**B**) After 5 weeks, the nude mice were executed and the tumor tissue was collected. (**C**) qRT-PCR was used to detect the relative expression of miR-146b-3p and E2F2 in tumor tissues of nude mice. (**D**) WB was used to detect the expression of E2F2 protein in tumor tissues of nude mice. (**E**) H&E staining of the lungs from mice. Scale bars, 50 μm. (**F**) qRT-PCR was used to detect the relative expression of circ_RPPH1 in tumor tissues of nude mice. ^*^indicates *P* < 0.05; ^**^indicates *P* < 0.01.

At the end of the study, we also established the xenotransplantation model of nude mice. After injecting of sh-circ_RPPH1, the volume of nude mice decreased evidently with the increase of time and the mass of tumor tissues collected after 5 weeks' death, and the volume of sh-circ_RPPH1 group was evidently reduced compared with that of sh-NC group (Figure 6A, 6B). qRT-PCR indicated that miR-146b-3p in nude mice tumor tissues increased evidently, while E2F2 decreased evidently (Figure 6C). WB test showed that E2F2 protein in nude mice tumor tissues decreased evidently (Figure 6D). Therefore, circ_RPPH1 might be applied as the therapeutic target for BC. Meanwhile, the lung metastasis of the mice was repressed by the depletion of circ_RPPH1 in the model (Figure 6E). The inhibition of circ_RPPH1 was validated in the mice (Figure 6F).

## DISCUSSION

At present, an increasing number of studies revealed that circRNA acts in the tumorigenicity of tumors, and it has caused concern in many fields [24]. circRNA is found differentially in various tumors such as lung carcinoma, [25] pancreatic carcinoma [26] and hepatocellular carcinoma [27]. We found in our tests that circ_RPPH1 was highly expressed in BC through chip screening, and it was expected to become a potential target of BC. Therefore, this study focused on the role of circ_RPPH1 in BC.

We first analyzed GSE101123 chip and found that circ_RPPH1 in BC increased evidently. Furthermore, the expression of circ_RPPH1 in BC patients showed the same trend by qRT-PCR, which suggested that circ_RPPH1 had a carcinoma-promoting effect in BC. We also found that patients with high circ_RPPH1 had higher probability of TNM high stage and lymphatic metastasis, which indicated that circ_RPPH1 might participate in the progression of BC. In order to further understand whether circ_RPPH1 is participate in the progression of BC, we established siRNA infected BC cells, and found that the proliferation, invasion and migration of BC cells were obviously hindered after transfection of si-circ_RPPH1#2, the cells were blocked in S phase, and the apoptosis was induced. These results indicated that circ_RPPH1 could hinder the growth and metastasis of BC cells by blocking cell cycle and inducing apoptosis, which might be a therapeutic target in clinic. Our finding provided the innovative function of circ_RPPH1 in BC and presented the crucial evidence of the crucial roles of circular RNAs in BC progression. It has been reported that circ_RPPH1 is enhanced in hepatocellular carcinoma, which can play a carcinogenic role by regulating miR-1296-5p [18]. Our data is consistent the previous study in which circ_RPPH1 serves as a contributor to the cancer progression. But we explored the different mechanism in the current study. There are still some limitations in this investigation. For example, we failed to analyze the survival outcomes of circRPPH1 in TCGA, GEO, or other database due to the information limitation. Unfortunately, we also could not assess the survival outcomes of circRPPH1 in our 44 clinical BC samples due to the limited follow-up time and we will analyze the survival outcomes of circRPPH1 in more BC samples in future investigations. We added the related discussion to the revised manuscript.

According to ceRNA theory, RNA transcripts, including mRNA, lncRNA, pseudogenes and circRNA, can cross-talk with each other and regulate their expression through competing and sharing miR response elements (MRE), thus establishing a new post-transcriptional regulatory network [28]. Moreover, studies revealed that circRNA can regulate the downstream target genes by acting as miR sponge. For example, [29] circTADA2As hinders the progression and metastasis of BC by targeting miR-203a-3p/SOCS3 axis. Other studies found that the enhanced circ_0079586 can predict the poor prognosis of glioma and accelerate its progression through the interaction with miR-183-5p/MDM4 [30]. In order to verify that circ_RPPH1 has the effect as ceRNA, we first localized it. Circ_RPPH1 is mainly located in the cytoplasm of BC cells by fluorescence *in situ* hybridization and subcellular grading, indicating that circ_RPPH1 has the effect as ceRNA. Then, we predicted the potential miR of circ_RPPH1 through the current website, and found a targeted binding site between circ_RPPH1 and miR-146b-3p. RIP, RNA pull-down and double luciferase report confirmed that circ_RPPH1 can directly interact with miR-146b-3p. In addition, qRT-PCR indicated that miR-146b-3p in BC patients was reduced, which had a negative correlation with circ_RPPH1. This also confirmed the targeting correlation. Our data indicates a novel function of miR-146b-3p in BC progression and elucidates the correlation of circ_RPPH1 with miR-146b-3p in BC cells. MiR-146b-3p may just one of the downstream miRNAs of circ_RPPH1 in the modulation of BC development and other potential miRNAs should be explored in the system.

As an important cyclin, E2F2 belongs to E2F transcription factor family and acts in cell cycle regulation and tumor suppressor proteins [31]. Many explorations indicated that E2F2 acts in regulating the occurrence and development of BC [32]. We also predicted the target gene of miR-146b-3p, and found that E2F2 and miR-146b-3p had a targeted binding relationship. Double luciferase report confirmed that miR-146b-3p can target E2F2, and qRT-PCR and WB also found that E2F2 changed in different degrees in BC cells after transfection with miR-146b-3p-mimics and anti-miR-146b-3p, suggesting that miR-146b-3p and E2F2 had targeted regulation. In addition, we also found that E2F2 was negatively correlated with miR-146b-3p, but positively correlated with circ_RPPH1, indicating that there was a regulatory correlation among circ_RPPH1, miR-146b-3p and E2F2. The well-known E2F2 regulatory pathway may be just one of the downstream mechanisms underlying circ_RPPH1-mediated BC progression and other mechanisms should be explored in future investigations.

In order to verify the speculation, we conducted a rescue experiment. It was found that the increase of proliferation of cells, the increase of invasion and migration, and the inhibition of apoptosis could be reversed by co-transfection of miR-146b-3p-mimics. Furthermore, qRT-PCR and WB experiments suggested that E2F2 mRNA and protein in cells enhanced obviously after transfection with pLCDH-circ_RPPH1, but there was no change in E2F2 mRNA and protein after co-transfection with miR-146b-3p-mimics compared with Vector. The results indicated that circ_RPPH1 can regulate miR-146b-3p/E2F2 axis. At the end of the study, we conducted an *in vivo* experiment, and injected the stably transfected sh-circ_RPPH1 BC cells into nude mice. It was found that knocking down circ_RPPH1 could hinder the growth of tumor volume and mass in nude mice, which was achieved by miR-146b-3p/E2F2 axis. Our finding provides new insight into the mechanism by which circ_RPPH1 contributes to BC progression by targeting miR-146b-3p/E2F2, indicating the unreported correlation of circ_RPPH1, miR-146b-3p and E2F2 in the development of BC cells. The E2F2 may just one of the targets of circ_RPPH1/miR-146b-3p signaling in the regulation of BC development and other potential targets need to be investigated in future studies.

This study suggests the related mechanism of circ_RPPH1 in BC, but still had some limitations. For example, circRNA has unique structure, high stability and specific expression mode, which may become a potential clinical biomarker. However, our study did not test the correlation of circ_RPPH1 with the long-term prognosis because of the short sample collection time. Meanwhile, tumor drug resistance causes the failure of clinical treatment at present, and it is not clear whether circ_RPPH1 can sensitize the tumor in BC drug resistance. Therefore, we hope to carry out more basic experiments and collect more clinical samples in future research to improve our research in future investigations.

To sum up, circ_RPPH1 in BC can promote the progression of BC through miR-146b-3p/E2F2 axis, and it might be a therapeutic target for clinical BC treatment.

## MATERIALS AND METHODS

### Analysis of gene expression omnibus (GEO) chip

We logged into GEO database and entered keywords (BC, circRNA) for search. According to the number of samples and the missing situation, GSE101123 chip was chosen for analysis. There were 8 tumor samples and 3 normal samples on GSE101123 chip. The Series Matrix File(s) and GPL19978 were downloaded and merged into a matrix file. In addition, the name of circrRNA was corrected by using VLOOKUP function. Then the difference circrRNA was analyzed using limma package, and volcano map and heat map were visualized, respectively.

### Clinical data

In this study, 44 patients with BC from January 2017 to January 2019 were obtained as the research participants. Tumor tissues and adjacent tissues were obtained during the operation and transported to the laboratory for testing. All patients in this study have signed informed consent, and none of them received radiotherapy and chemotherapy before the test. This study conformed to the Ethics Committee of the First Affiliated Hospital of Shandong First Medical University and the Declaration of Helsinki [33].

### Cell culture

BC cells (MCF-7, MDA-MB-231, MDA-MB-468, and MDA-MB-453) and human normal mammary epithelial cells (MCF-10A) were all from ATCC, and BC cells were cultivated in DMEM. MCF-10A cells were cultivated in RPMI-1640 medium, and all the above tests were carried out at 37°C with 5% CO_2_.

### Cell transfection

The specific knockdown of circ_RPPH1 (si-circ_RPPH1#1-3) was realized by using three siRNA oligonucleotides targeting post splicing junctions, which were synthesized by Ribobio (China). To over-express circ_RPPH1, the full-length cDNA was amplified in 293 *T* cells, and then cloned into pLCDH-circ_RPPH1 (Geneseed, China) containing front and back circular frames, which did not apply circ_RPPH1 sequence as a vector. miR-146b-3p-mimcs, anti-miR-146b-3p (miR-146b-3p inhibitor) and/or miR-NC were synthesized by Genepharma (China). Lipofectamine 3000 was applied to transfect BC cells.

### qRT-PCR detection

Total RNA was isolated from serum and tissues using TRIzol (Takara, Dalian, China) according to the manufacturer’s instructions. The integrity and purity of the extracted total RNA were measured using NanoDrop One (Thermo Fisher Scientific, Waltham, MA, USA) ultra-micro UV spectrophotometer. Reverse transcription was performed using the PrimeScript RT reagent Kit (Takara, Dalian, China) with gDNA Eraser. After removing the genomic DNA at 42°C for 2 min, the tissue RNA was reverse transcribed into cDNA under the following conditions: 37°C for 15 min and 85°C for 5 s. Serum RNA was reverse transcribed into cDNA using a RevertAid H Minus First Strand cDNA Synthesis Kit (Thermo Fisher Scientific, Waltham, MA, USA) under the following conditions: 25°C for 5 min, 42°C for 60 min, and 70°C for 5 min. The product was immediately stored at −80°C until use. ABI 7500 PCR system was applied for analysis. The qRT-PCR reaction was performed 95°C for 5 min, followed by 40 cycles of 95°C for 10 s and a primer-specific annealing temperature of 60°C for 30 s. The relative quantification values for RNA were calculated by the 2^−ΔΔCt^ method using GAPDH as an internal reference. circ_RPPH1 primer sequence: upstream primer 5′-AGCTTCGGGGAGGTGAGTT-3′, downstream primer 5′-TGGCCCTAGTCTCAGACCTT-3′; miR-146b-3p primer sequence: upstream primer 5′-ACACTCCAGCTGGGGGTCTTGACTCAGGTG--3′, downstream primer 5′-CTCAACTGGTGTCGTGGAGTCGGCAATTCAGTTGAGACGGGACA3′; E2F2 primer sequence: upstream primer 5′-AAGTGCATCAGAGTGGATGGCCT-3-3′, downstream primer 5′-AATGAACTTCTTGGTGAGCAGCCC-3′; GAPDH primer sequence: upstream primer 5′-GAATGGGCAGCCGTTAGGAA-3′, downstream primer 5′-AAAAGCATCACCCGGAGGAG-3′; U6 primer sequence: upstream primer 5′-CTCGCTTCGGCAGCACA-3′, downstream primer 5′-AACGCTTCACGAATTTGCGT-3′.

### Detection of cell activity

The proliferation was tested using CCK-8. The steps were described as follows. Transfected BC cells were obtained and cultivated in 96-well plate with 2 × 10^3^ cells/ well. Altogether 100 μL serum-free medium and 10 μL CCK-8 solution were put into the well, and then cultivated at 37°C for 1h. The absorbance was tested using a microplate reader (Bio-Rad, Hercules, CA, USA).

### Detection of cell invasion

The invasion and migration were tested using Transwell method. Matrigel was not pre-coated in Transwell for migration test (BD falcon) but for invasion detection. The specific steps were as follows. The transfected BC cells (1 × 10^5^ cells) were collected, cultivated in 200μL serum-free medium, and then added into Transwell upper chamber. The lower chamber contains a medium with 20% FBS as a chemical attractant. After 24–48 hours, the cells on the lower surface were fixed with methanol, dyed with 0.1% crystal violet, and then photographed.

### Detection of cell apoptosis and cycle

The apoptosis was determined using annexin V-FITC (Invitrogen). The transfected BC cells were digested and rinsed with cold PBS. Cell resuspension was adjusted to 1 × 10^6^ cells/ml in 100μL binding buffer of annexin V. PI was added and cultivated in dark for 20 minutes. The apoptotic cells were tested using flow cytometry (BD Biosciences). For cell cycle determination, the transfected BC cells were rinsed with PBS three times and fixed with 80% ethanol. Then, the cells were cultivated with RNase A and 20μg/ ml of PI for 20 minutes. Cell cycle was tested the same way as above.

### Fluorescence *in situ* hybridization

A specific circ_RPPH1 FISH probe labeled with Cy3 (Geneseed, China) was designed and used in the experiment. Cells attached to slides were immobilized with 4% paraformaldehyde, washed with PBS, and then digested by protease K (Sangon, Shanghai, China) at 37°C for 5 min. After washing with PBS, the cells were immobilized with 1% paraformaldehyde followed by successive dehydration in 70, 85, and 100% alcohol. Hybridization solution dilute probe was dripped onto the cell slide followed by denaturation at 73°C for 3 min and hybridization overnight at 60°C in the dark. The slides were then washed with 50% formamide/2 × SSC preheated to 43°C, 0.1% NP-40/2 × SSC preheated to 37°C, and DAPI staining solution at room temperature. Images were acquired using a laser confocal microscope (Leica, Mannheim, Germany).

### Actinomycin D and RNase R treatment assay

To compare the stability of linear RNA and circRNA, Actinomycin D (MedChemExpress, China) was added into medium to block RNA transcription and the solvent dimethyl sulfoxide (DMSO; Sigma) was applied as a negative control. Cells were treated with actinomycin D or DMSO in a final concentration of 1 μg/mL for 0, 4, 8, 12, and 24 h, and then the RNA was extracted for RT-qPCR detection, using 18S as an internal reference. For RNase R treatment, 2 mg total RNA was incubated for 15 min at 37°C with or without 3 U/mg RNase R (No. R0301, Geneseed, China), and followed by RT-qPCR analysis.

### Determination of subcellular grading

In this study, PARIS™ kit (Ambion, Austin, TX, USA) was used to locate circRNA in cells. The methods were as follows. Subcellular grading was performed in 1 × 10^4^ cells. First, the collected cells were re-suspended with cell separation buffer. Then, the cells were placed on ice for ten minutes. After centrifugation, cell destruction buffer was used to preserve nuclear precipitate and supernatant to extract RNA. Finally, the cells were quantified by qRT-PCR with GAPDH and U6 as internal references.

### RNA immunoprecipitation (RIP)

RIP experiment was performed using Magna RIP kit, and the experiment was carried out according to the instructions of the kit. The steps were as follows. miR-146b-3p-mimcs or miR-NC BC cells were collected after transfection for 48 hours. Lysis was carried out in NA lysis buffer, and then the cell lysate was cultivated with magnetic beads, which were coupled with AGO2 or negative control IgG antibody at 4°C for 4 h. The beads were then rinsed with washing buffer. Then immunoprecipitated RNA and protein were purified, and target RNA and AGO2 were detected by qRT-PCR and Western blot. All the above products were obtained from Millipore.

### RNA pulldown

Biotin-labeled circ_RPPH1 were synthesized by QIAGENE, and transfected to BC cells for 48 hours. The cells were then lysed and hatched with magnetic beads (Thermo) for 3 hours, followed by washing and detection by real-time PCR to measure the level of the indicated miRNAs.

### Double luciferase report

The targeting correlation among circRNA, mRNA and miR was tested. The 293T cells (5 × 10^4^) were inoculated in a 24-well plate. circ_RPPH1-WT, circ_RPPH1-MUT, E2F2-WT, and E2F2-MUT were co-transfected with miR-146b-3p-mimics and miR-NC respectively, and then the cells were cultivated for 24 h. The cultivated cells were collected and tested to determine the fluorescence activity.

### Western blot

The transfected cells and the total protein were obtained by RIPA buffer, and the protein was quantified using BCA kit. A total of 10% sodium dodecyl sulfate polyacrylamide gel electrophoresis (SDS-PAGE) was applied for separation and then moved to polyvinylidene fluoride (PVDF) membrane, which was cultivated with primary antibody (1:1000, anti-E2F2) at 4°C and with 5% skim milk in the dark, and then the secondary antibody labeled with horseradish peroxidase (HRP) was added, and the immune complex was tested by ECL Western Blotting Kit. The relative protein was tested using Image- Pro plus 6.0, and β -actin was applied as the internal reference.

### Xenotransplantation model of nude mice

The animal study was conducted and conformed to the First Affiliated Hospital of Shandong First Medical University. The experiment was conducted according to the Laboratory animal—Guideline for ethical review of animal welfare. Six five-week-old female BALB/c nude mice were selected and grouped into two groups, which were injected subcutaneously with stable transfected MDA-MB-231 cells (5 × 10^6^) sh-circ or sh-NC lentiviral vector (FulenGen). The experiment was lasted for 35 days, and the tumor volume was calculated every 7 days (Width^2^ × length/2). The nude mice were euthanized (inhaled with carbon dioxide) 35 days later, and then the tumor tissues were obtained and the contents of circ_RPPH1, miR-146b-3p and E2F2 were tested. Lung metastases were analyzed by gross examination of freshly dissected lungs and histopathological review of hematoxylin and eosin (H&E)-stained lung sections.

### Statistical analysis

Graphpad Prism 6.0 and SPSS 20.0 were applied for data analysis, independent sample *t* test for pari-group comparison. Counting data were represented in percentage (%) and compared by chi-square test. Single factor analysis of variance was applied for multi-group comparison, and LSD-t test for post-event comparison. The expression profiles at different time points were verified by repeated measurement analysis of variance (represented as F), and the back testing was completed by Bonferroni. Pearson test was applied to explore the correlation among genes. *P* < 0.05 indicates statistically significant.

## Supplementary Materials

Supplementary Figures
