# Supplementary material for: Mediation of circ_RPPH1 on miR-146b-3p/E2F2 pathway to hinder the growth and metastasis of breast carcinoma cells
Source: Aging (Albany NY). 2021 Aug 25;13(16):20552–68. doi: 10.18632/aging.203439 (PMC8436922; doi:10.18632/aging.203439)
Supplement: Supplementary Figures [file aging-13-203439-s001.pdf]

SUPPLEMENTARY FIGURES

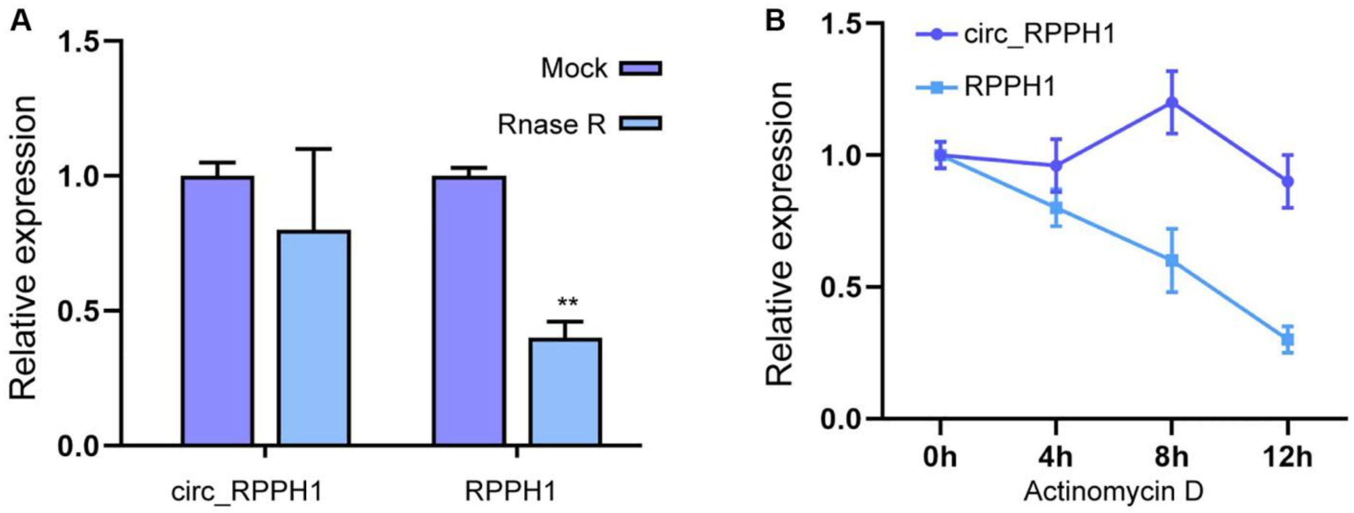

Supplementary Figure 1. Characterization of circ_RPPH1 in BC. (**A**) qRT-PCR was used to detect the relative expression of circ_RPPH1 in BC cells treated with RNase R. (**B**) qRT-PCR was used to detect the relative expression of circ_RPPH1 in BC cells treated with Actinomycin D. **indicates *P* < 0.01.

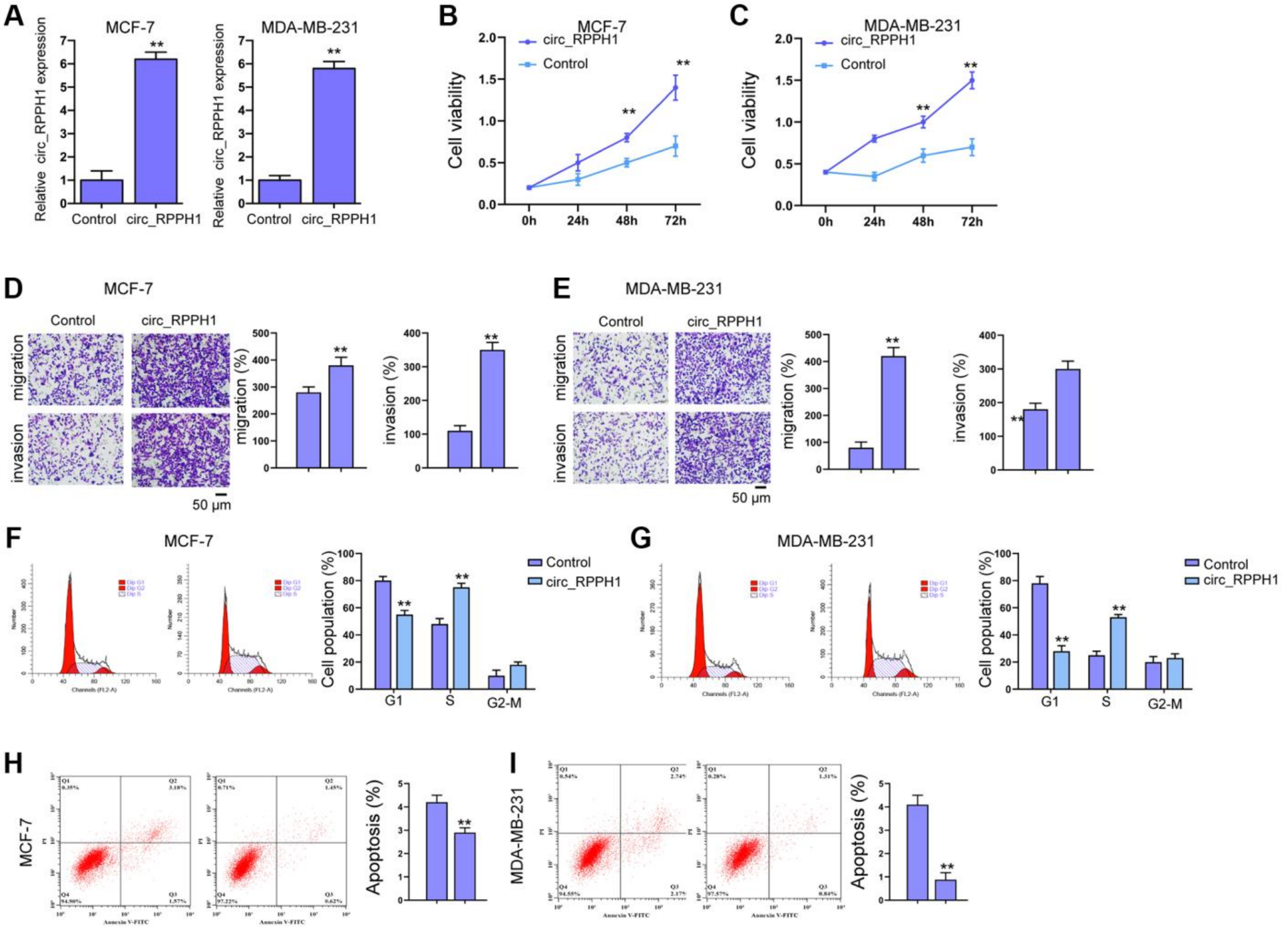

Supplementary Figure 2. Carcinogenic role of circ_RPPH1 in BC. (**A**). qRT-PCR was used to detect the relative expression of circ_RPPH1 in BC cells treated with circ_RPPH1 overexpressing plasmid. (**B**, **C**) CCK-8 test was used to detect the proliferation of BC cells transfected with treated with circ_RPPH1 overexpressing plasmid. (**D**, **E**) Transwell test showed the invasion and migration of BC cells treated with circ_RPPH1 overexpressing plasmid. Scale bars, 50 μm. (**F**–**I**) The changes of cell cycle and apoptosis rate of BC cells treated with circ_RPPH1 overexpressing plasmid were detected by flow cytometry. *indicates $P < 0.05$; **indicates $P < 0.01$.

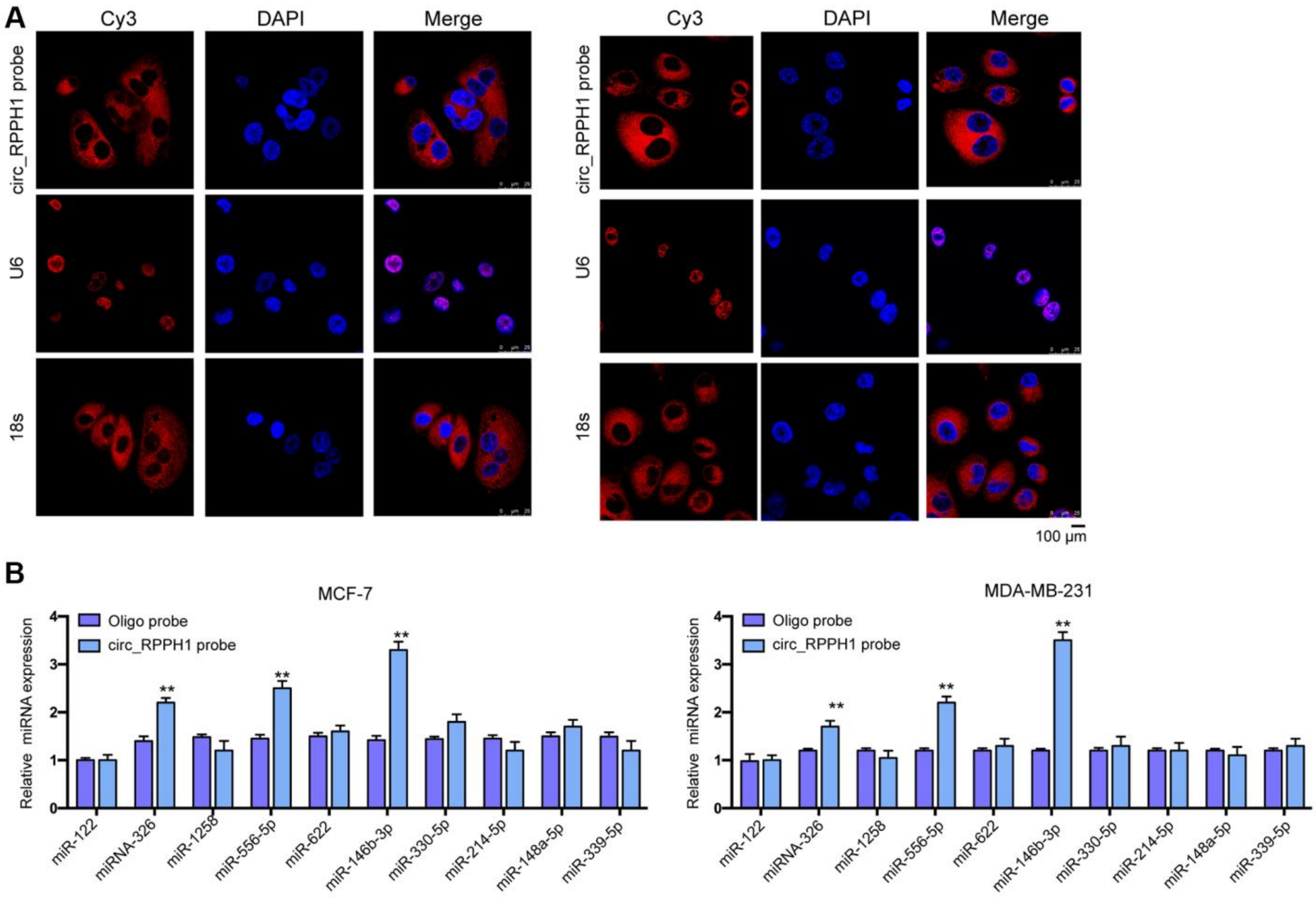

Supplementary Figure 3. circ_RPPH1 acts as miR-146b-3p sponge for regulation. (**A**) Subcellular localization analysis using FISH assays of circ_RPPH1 distribution in MCF-7 and MDA-MB-231 cells. Scale bars, 100 μm. (**B**) RNA pull down was used to analyze the interaction of circ_RPPH1 with the indicated miRNAs in MCF-7 and MDA-MB-231 cells. *indicates $P < 0.05$; **indicates $P < 0.01$.
